# Immunopathological Aspects of Experimental *Trypanosoma cruzi* Reinfections

**DOI:** 10.1155/2014/648715

**Published:** 2014-06-24

**Authors:** Juliana Reis Machado, Marcos Vinícius Silva, Diego Costa Borges, Crislaine Aparecida da Silva, Luis Eduardo Ramirez, Marlene Antônia dos Reis, Lúcio Roberto Castellano, Virmondes Rodrigues, Denise Bertulucci Rocha Rodrigues

**Affiliations:** ^1^Laboratory of Immunology, Federal University of Triângulo Mineiro, Cefores, Frei Paulino Street, 30, 38025-180 Uberaba, MG, Brazil; ^2^Discipline of Cell Biology, Federal University of Triângulo Mineiro, Frei Paulino Street, 30, 38025-180 Uberaba, MG, Brazil; ^3^Discipline of Parasitology, Federal University of Triângulo Mineiro, Frei Paulino Street, 30, 38025-180 Uberaba, MG, Brazil; ^4^Discipline of Pathology, Federal University of Triângulo Mineiro, Frei Paulino Street, 30, 38025-180 Uberaba, MG, Brazil; ^5^Technical School of Health, Federal University of Paraíba, 58051-900 João Pessoa, PB, Brazil

## Abstract

Chagas disease is caused by *Trypanosoma cruzi* infection. Besides the host-related factors, such as immune response and genetic background, the parasite, strain, and occurrences of reinfection episodes, may influence disease outcome. Our results demonstrate that both the primary infection and the reinfection with the Colombiana strain are connected with lower survival rate of the mice. After reinfection, parasitaemia is approximately ten times lower than in primary infected animals. Only Colombiana, Colombiana/Colombiana, and Y/Colombiana groups presented amastigote nests in cardiac tissue. Moreover, the mice infected and/or reinfected with the Colombiana strain had more *T. cruzi* nests, more intense inflammatory infiltrate, and higher *in situ* expression of TNF-*α* and IFN-*γ* than Y strain. Antigen-stimulated spleen cells from infected and/or reinfected animals produced higher levels of TNF-*α*, IFN-*γ*, and IL-10. Our results reinforce the idea that Chagas disease outcome is influenced by the strain of the infective parasite, being differentially modulated during reinfection episodes. It highlights the need of control strategies involving parasite strain characterization in endemic areas for Chagas disease.

## 1. Introduction

Chagas disease is caused by* Trypanosoma cruzi (T. cruzi)* infection. Nowadays there are approximately ten million people infected worldwide, especially in Latin America [1].* Trypanosoma cruzi* is genetically diverse being group on strains or subspecies with peculiar behavior. Among them, Y and Colombiana are referred to as polar strains owing to their morphological aspects, tissue tropism, and kinetics of parasitaemia [2, 3]. Several strains may be circulating in the same endemic area, where individuals might be prone to multiple exposures to the parasite.

The acute phase of Chagas disease is characterized by high parasitaemia with excessive activation of the immune system. It includes elevated plasma levels of Th1-type cytokines (mainly TNF-*α* and IFN-*γ*) associated with resistance to parasite infection, as well as strong activation of T and B cells and severe inflammatory processes connected with the parasitism. This phase is followed by the development of acquired immunity, leading to the control of parasitaemia and tissue parasitism. On the other hand, the chronic phase of the disease is usually marked by low parasitaemia and tissue parasitism but high levels of antibodies [4, 5]. The presence of Th1 cytokines in this phase appears to be connected with the severe forms of the disease [6, 7], whereas IL-10 seems to protect the host by promoting less tissue damage [8–10]. In human disease, morphological changes are characterized by mononuclear inflammatory infiltrate and fibrotic areas in this phase [11].

Earliest studies about the role of reinfections in Chagas disease progression pointed to the development of resistance after the first infection, culminating to a mild acute phase [12–14]. Nevertheless, more recent studies indicate that reinfections may lead to the development of severe forms of the disease [15–18], whereas other authors could not observe any relation in experimentally infected dogs [19]. Controversies about the role of reinfections in the course of Chagas disease can be better clarified through histopathological evaluation and measurement of cytokine production against different* T. cruzi* strains.

## 2. Materials and Methods

### 2.1. Animals

Male C57BL/6 mice (8–10 weeks old) were obtained and housed in the animal facility of UFTM, Uberaba, Brazil. Mice were given water and food ad libitum during the experimental period and all procedures were approved by the local ethical committee for animal research (CEUA—protocol number 176).

### 2.2. Infection and Parasitaemia

For the present study we used 75 mice, of which 40 were subcutaneously infected with 3,000 forms of the Colombiana strain and 30 were infected with the Y strain. Five uninfected animals were kept as control group. The 70 infected animals were observed for 90 days until they reached the chronic phase of Chagas disease. During this time, two animals infected with the Y strain died, and 14 animals in the group infected with the Colombiana strain died.

Ninety days after primary infection of the animals with the Colombiana strain, twelve animals were reinfected with 3,000 forms of trypomastigotes of the Colombiana strain (Col/Col), eight were reinfected with the Y (Col/Y) strain, and six were not reinfected (Col). Amongst the animals infected with the Y strain, ten were reinfected with the Y strain (Y/Y), ten were reinfected with the Colombiana (Y/Col) strain, and eight were not reinfected (Y).

We performed a direct parasitological examination to look for trypomastigotes in the infected animals on Days 7, 14, and 21 after primary infection. After the reinfection, the animals were reinfected and parasitaemia was performed again on Days 7, 14, and 21 in accordance with protocol [20]. Euthanasia was performed on day 111° in mice infected with Y and Col strains, whereas reinfected animals from groups Y/Y, Y/Col, Col/Col, Col/Y were euthanasied on day 21 after reinfection. The procedure was performed in CO_2_ chamber. Blood was collected and autopsy was subsequently performed in order to collect the spleen for* in vitro* cell culture and the heart for* in situ* immunohistochemistry. The other organs of interest were collected and stored for further analysis.

### 2.3. Histological Analysis

#### 2.3.1. Inflammatory Infiltrate

For inflammatory infiltrate analysis we used hematoxylin and eosin (HE) stained slides of cardiac tissue (ventricle). Qualitative analysis of infiltrate was performed so as to classify the type of infiltrate as predominantly mononuclear (macrophages and lymphocytes) or polymorphonuclear (neutrophils and eosinophils). The cellular type observed in more than 50% of the infiltrate was regarded as prevalent. Semiquantitative analysis of infiltrate was also performed, and inflammatory infiltrate was classified as follows: mild (involvement > 25% of the tissue), moderate (25%–50% of the tissue), or severe (involvement > 50% of the tissue).

#### 2.3.2. Immunohistochemistry for Detection of* T. cruzi* Nests

Ventricular tissue sections were fixed in formaldehyde for immunohistochemistry, and endogenous peroxidase blocking was performed using 3% H_2_O_2_ in methanol. Then, rabbit anti-*T. cruzi* antibody (1 : 250) (in house) was added at room temperature for 2 hours. Then, the slides were incubated with peroxidase-conjugated protein A (1 : 500) for 2 hours. To reveal the reaction we used H_2_O_2_ (0.05%) and 1 mg/mL DAB (1,4-dideoxy-1,4-imino-D-arabinitol-diaminobenzidine) (sigma Chemical Co., St Louis, MO, USA) in tris-HCl buffer (pH 7.4). The sections were counterstained with hematoxylin and analyzed using a common light microscope. Heart parasitism was quantitatively evaluated according to the presence or absence of amastigote nests.

#### 2.3.3. Quantification of Fibrosis

We carried out a morphometric evaluation of fibrous conjunctive tissue in heart sections stained with Sirius Red. The slides were analyzed using digital morphometry in polarized light microscope at a final magnification of ×400. Morphometry was performed using KS300 Imaging System (Carl Zeiss). Fibrosis was quantified along the length of the histological section and expressed in percentage of affected tissue.

### 2.4. Immunological Analysis

#### 2.4.1. Spleen Cell Culture

Spleens of mice were collected and maintained in RPMI 1640 medium (*GE* Health care, Uppsala, Sweden) and macerated for individualization of cells. These suspended cells were washed three times by centrifugation at 400 ×g for 15 min at 8°C in RPMI 1640. Then, they were counted in a Neubauer chamber and resuspended to 2 × 10^6^ cells/mL in RPMI 1640 medium with addition of 50 mM Hepes (Gibco, Grand Island, NY, USA), 5% of inactivated fetal bovine serum (GIBCO-US), 2 mM L-glutamine (GIBCO-US), 0.05 mM 2*β*-mercaptoethanol (GIBCO-US), and 40 *μ*g/mL gentamicin (NEOQUÍMICA, Anápolis, GO, BR). Then, 2 × 10^6^ cells were incubated without stimulus and with 5 *μ*g/mL of* T. cruzi* antigen in 24-well culture plates (BD Pharmingen, San Diego, CA, USA). The cultures were kept in a moist incubator with 5% CO_2_ at 37°C for 24 and 72 hours. The supernatants were collected and maintained at −70°C until analysis.

#### 2.4.2. Preparation of Cardiac Tissue Homogenate

Heart tissue sections were immersed in PBS solution containing complete protease inhibitor (Sigma, St. Louis, MO, USA) and Nonidet-P40. After that, they were submitted to tissue homogenizer. The homogenate obtained was centrifuged at 14000 ×g for 10 minutes and the supernatant was maintained for quantification of cytokines and total proteins.

#### 2.4.3. Quantification of Cytokines in Supernatants of Spleen Cell Culture and in Cardiac Tissue Homogenate Using CBA

Cytokines IL-2, IL-4, IL-5, IL-10, IL-12p70, IL-17, TNF-*α*, and IFN-*γ* were quantified using Cytometric Bead Array—CBA (BD Pharmingen, San Diego, CA, USA) in accordance with the manufacturer's specifications. The samples and the recombinant cytokines were incubated with beads with different fluorescence intensities conjugated with specific capture antibody for each cytokine of interest. After incubation, the beads were washed with saline solution and analyzed in BD FACS CALIBUR flow cytometer, using CellQuest software. Upon data acquisition of samples and of recombinant cytokines, they were analyzed using FCAP Array v2.0 software (Soft Flow, USA) and the concentrations of the cytokines were measured by comparison to the standard curve. The concentrations of cytokines in cardiac tissue homogenate were normalized based upon the concentration of total proteins in each homogenate and were quantified using the Micro-Lowry method in accordance with the manufacturer's instructions (Pierce, Rockford, IL, USA).

### 2.5. Statistical Analyses

GraphPad Prism 5.0 software (GraphPad Software, USA) was used. Mann-Whitney test (*U*) was used for analysis between two groups, and for analysis among more than two groups ANOVA test (*F*) was used for data with normal distribution and Kruskal Wallis test (*H*) was used for data with nonnormal distribution. Qualitative variables were expressed as percentage and the associations between them were analyzed using the chi-square (*χ* ^2^) test. Survival rate analyses were performed using Log-Rank test. Results were considered statistically significant when *P* < 0.05.

## 3. Results

### 3.1. Survival Rate

**Figure 1:**
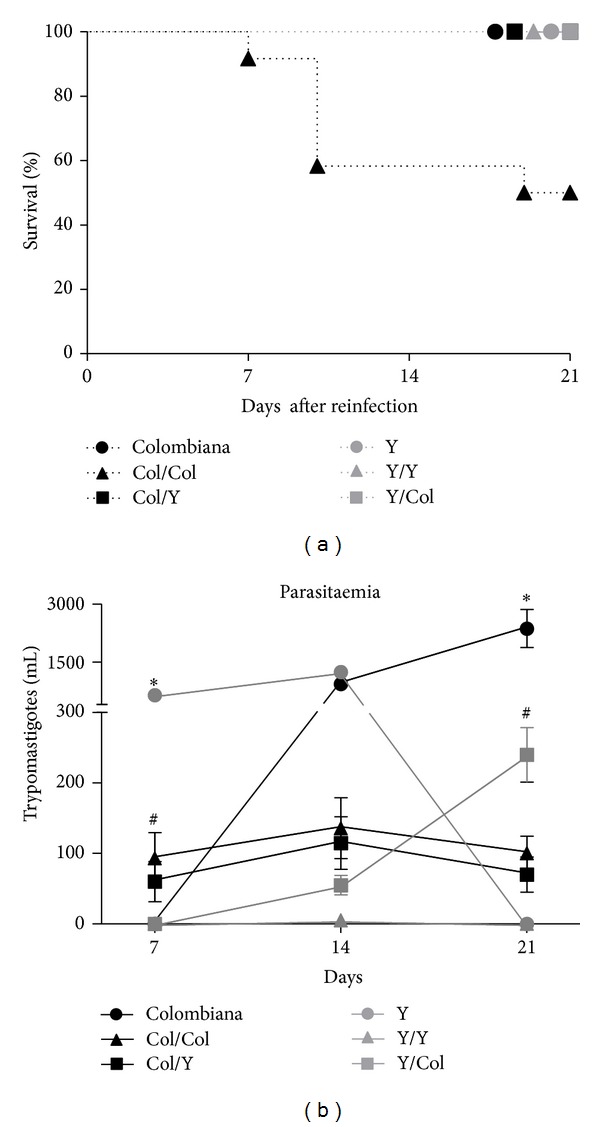
(a) Survival curve of reinfected mice, *P* = 0.0002, Log-rank test. (b) Parasitaemia of mice that were primarily infected and reinfected with the Colombiana and Y strains of* T. cruzi*. Parasitaemia levels were evaluated by counting the number of parasites in 5 *μ*L of blood collected from the tail vein. The symbols of each group represent the mean. *Statistical differences in animals primarily infected with the Colombiana strain or Y strain were analyzed on Days 7, 14, and 21; Mann-Whitney test. ^#^Statistical differences among the reinfected groups (Y/Y, Y/Col, Col/Col, Col/Y) were analyzed on Days 7, 14, and 21; Kruskal-Wallis test.

We analyzed the survival rate in animals primarily infected with the Y and Colombiana strains and in reinfected animals. During chronification of the disease, a survival rate of 93.3% (28/30 animals) was observed in the animals primarily infected with the Y strain. Among the animals primarily infected with the Colombiana strain we observed a survival rate of only 65% (26/40 animals) (*P* = 0.0055, Log-rank test). After reinfection, the group infected with the Colombiana strain and reinfected with the same strain had a mortality rate of 50% (6/12) (*P* = 0.0002, Log-rank test). In the remaining groups we did not observe mortality within the period of 21 days after reinfection (Figure 1(a)).

### 3.2. Parasitaemia

The animals primarily infected with the Y strain but not with Colombiana strain had detectable parasitaemia since Day 7 of infection (*P* = 0.012; *U* = 0.000). On Day 14 we did not observe any difference in parasitaemia among the primary infected groups (*P* = 0.139; *U* = 12.50). Nonetheless, on Day 21 increased parasitaemia was observed in the animals infected with the Colombiana strain (*P* = 0.0006; *U* = 0.000), whereas parasitemia in animals infected with the Y strain was not detected (Figure 1(b)).

After reinfection, the levels of parasitaemia were approximately ten times lower than in primary infected animals. Parasitaemia in animals infected with the Y strain and reinfected with the Y or Colombiana strains was significantly lower than parasitaemia of animals infected with the Colombiana strain and reinfected with the Y or Colombiana strains on Day 7 of reinfection (*P* = 0.035; *H* = 8.567). On Day 14 only the parasitaemia in Y/Y group was significantly lower than the levels of parasitaemia in the other reinfected groups (*P* = 0.008; *H* = 11.594). On Day 21 the levels of parasitaemia in Col/Col and Col/Y Groups were still similar to the previous days; however, Y/Col group had significantly higher levels of parasitaemia than the other groups, and Y/Y group had significantly lower levels than the other groups (*P* < 0.0001; *H* = 23.331) (Figure 1(b)).

### 3.3. Histopathological Analysis

**Figure 2:**
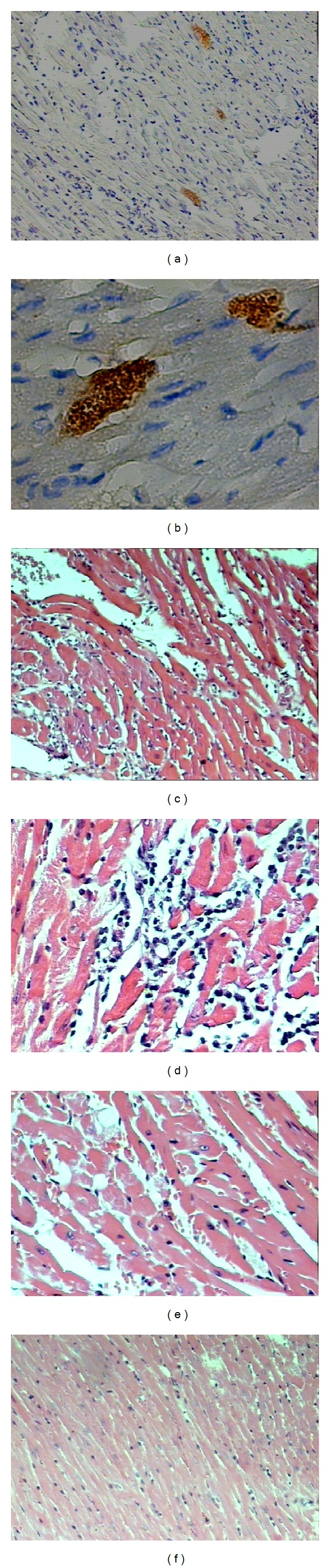
Histological heart sections of C57BL/6 mice. (a) Immunohistochemistry showing a* T. cruzi* nests in the animals infected and reinfected with the Colombiana strain (10x). (b) At a higher magnification, we noticed the details of* T. cruzi* nest in this same group (40x). (c) Inflammatory infiltrate in an animal infected with the Colombiana strain (HE, 10x). (d) Inflammatory infiltrate in the group infected and/or reinfected with the Colombiana strain (HE, 20x). (e) Inflammatory infiltrate in the group infected with the Y strain (HE, 10x). (f) Inflammatory infiltrate in the group infected and reinfected with the Y strain (HE, 10x).

Only Col, Col/Col, and Y/Col groups showed amastigote nests in cardiac tissue. Moreover, Col/Col group had a significantly higher percentage of animals with* T. cruzi* nests (*P* < 0.0001, *χ* ^2^ = 97.56) (Table 1, Figures 2(a)-2(b)).

The inflammatory infiltrate was predominantly mononuclear (Figures 2(e)-2(f)). The groups primarily infected with the Y strain showed a mild inflammatory infiltrate, except for Y/Col group, which showed a moderate inflammatory infiltrate. The groups infected and/or reinfected with the Colombiana strain showed a moderate-to-severe inflammatory infiltrate. Col/Col group showed a particularly severe inflammatory infiltrate in more than 60% of the animals (*P* < 0.0001, *χ* ^2^ = 273.8) (Table 1, Figures 2(c)-2(d)), and there was a worsening in comparison with the primary infection with the Colombiana strain.

We did not observe significant intensity of fibrosis among the groups primarily infected with the Colombiana strain and their subsequent reinfections and between these groups and the uninfected control group (*P* = 0.161; *F* = 1.94). Furthermore, we did not find significant differences among the groups primarily infected with the Y strain and their subsequent reinfections and between these groups and the uninfected control group (*P* = 0.066; *F* = 2.82) (Table 1).

### 3.4. Immunological Analysis

#### 3.4.1. Production of TNF-*α*, IFN-*γ*, and IL-10 in Cardiac Tissue

**Figure 3:**
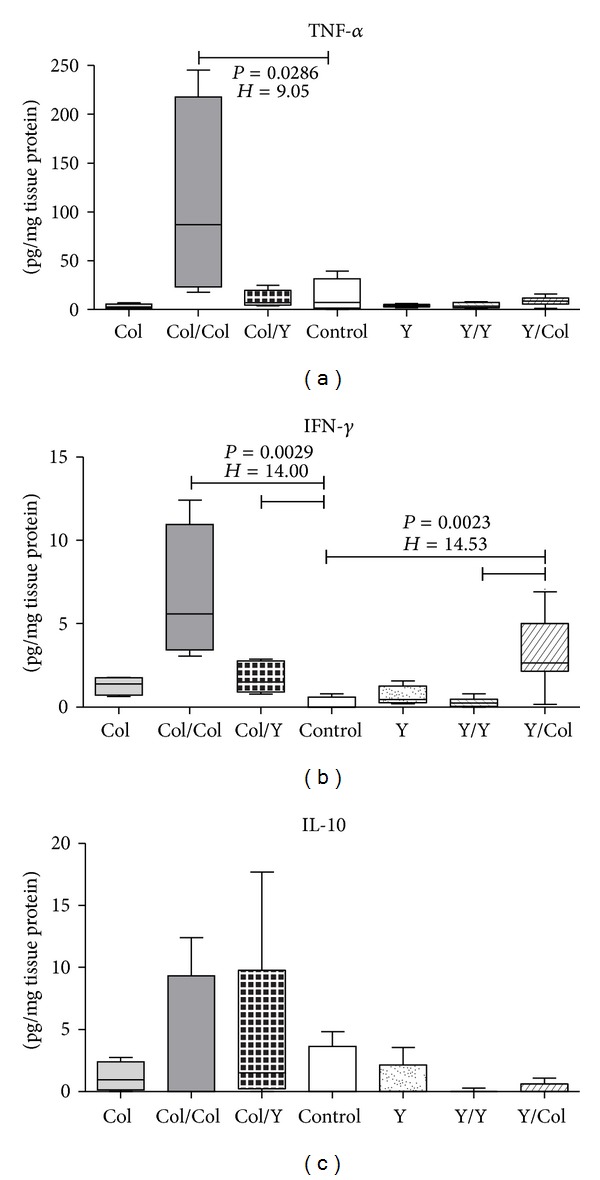
Production of TNF-*α*, IFN-*γ*, and IL-10 (pg/mL) in cardiac tissue of control mice infected and reinfected with* T. cruzi*. Kruskal-Wallis test followed by Dunn's multiple comparison test. Horizontal lines represent the median, bars represent 25–75 percentiles, and vertical lines represent 10–90 percentiles.

The expression of TNF-*α* was significantly higher in Col/Col group than in the control group. Although not significant, Col/Col group proved to produce more TNF-*α* than the other groups, especially the groups primarily infected with the Y strain. The groups primarily infected with the Y strain did not have a significant difference in TNF-*α* production in cardiac tissue (Figure 3(a)).

The expression of IFN-*γ* in cardiac tissue was significantly higher in Col/Col group and in Col/Y group than in the control group and higher in Y/Col group than in the control group and in Y/Y group. Although not significant, the groups infected or reinfected with the Colombiana strain proved to produce more IFN-*γ* than the groups infected with the Y strain and/or reinfected with the same strain, whose IFN-*γ* production was lower than the other groups (Figure 3(b)).

Both the groups primarily infected with the Colombiana strain and with the Y strain did not have a significant difference in IL-10 expression (Figure 3(c)). The expression of IL-4, IL-5, and IL-17 in cardiac tissue was decreased and did not have a significant difference among the groups (data not shown).

#### 3.4.2. Production of Cytokines in Supernatants of Spleen Cell Culture

**Figure 4:**
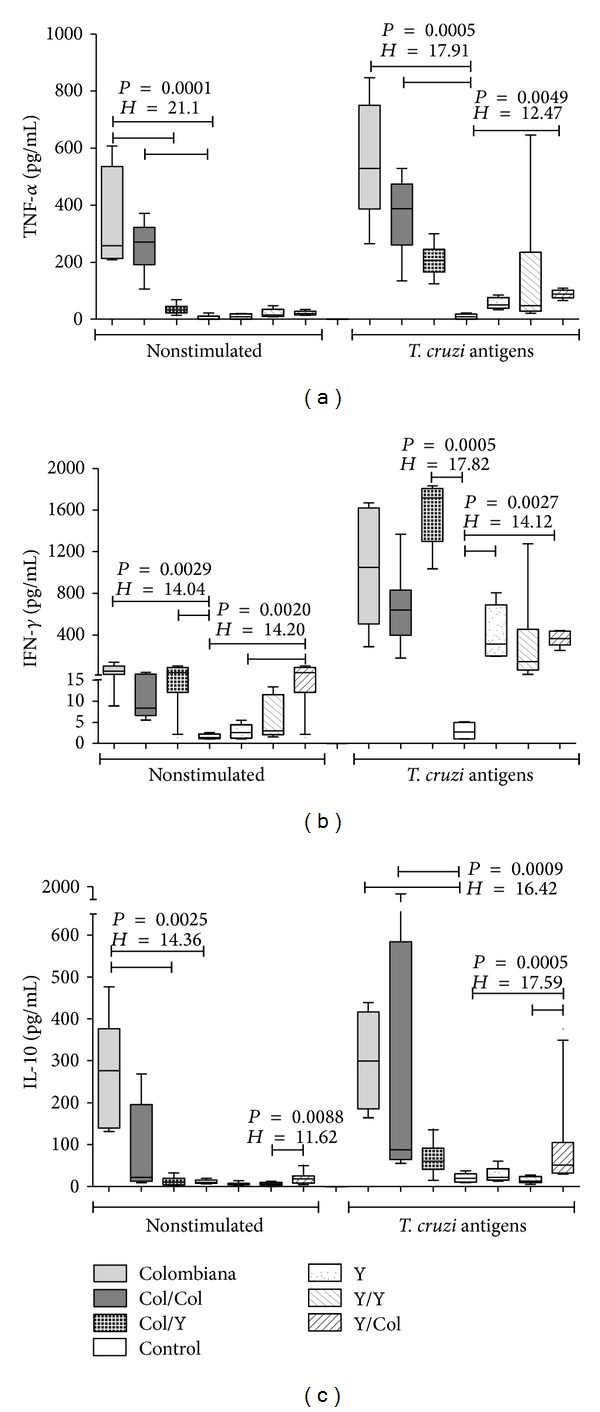
Levels of TNF-*α*, IFN-*γ*, and IL-10 (pg/mL) in supernatants of spleen cell culture of control mice and of infected and reinfected mice, unstimulated or stimulated with* T. cruzi* antigens. Kruskal-Wallis test followed by Dunn's multiple comparison test. Horizontal lines represent the median, bars represent 25–75 percentiles, and vertical lines represent 10–90 percentiles.

TNF-*α* levels were significantly higher in Col/Col group and in Col group than in the control group, in both unstimulated culture and stimulated cultures, and Col group had a significant increase in unstimulated culture in comparison with Col/Y group. Also, Y/Col mice in stimulated culture had significantly higher levels of TNF-*α* than the control group. Just like in the case of cardiac tissue, the groups primarily infected with the Colombiana strain had higher levels of TNF-*α*, both basal and antigen-specific (Figure 4(a)).

The production of IFN-*γ* was significantly higher in Col/Y group than in the control group, in both unstimulated and stimulated cultures. In unstimulated culture, Col group also had significantly higher levels of IFN-*γ* than the control group. Among the groups primarily infected with the Y strain, Y/Col group had significantly higher levels of IFN-*γ* than those of the control group, in both unstimulated and stimulated cultures. In unstimulated culture, Y/Col group had significantly higher levels than Y/Y group, as well as Y group had significantly higher levels of IFN-*γ* than the control group. Just like in the case of tissue production, the mice infected and/or reinfected with the Colombiana strain were the best producers of IFN-*γ* in unstimulated culture. Nevertheless, all the groups substantially increased IFN-*γ* production in stimulated culture (Figure 4(b)).

The production of IL-10 was significantly higher in Col group than in the control group, in both unstimulated and stimulated cultures. Col group also had significantly higher levels of IL-10 in unstimulated culture than Col/Y group, and Col/Col group had significantly higher levels in stimulated culture than the control group. We observed a significant increase in the production of IL-10 in Y/Col group in comparison with Y/Y group, in both unstimulated and stimulated cultures. Y/Col group also had significantly higher levels of IL-10 in the culture stimulated with* T. cruzi* than the control group. In general, the groups infected and/or reinfected with the Colombiana strain seem to produce more IL-10, especially in stimulated culture.

## 4. Discussion

At the onset* T. cruzi* infection it is possible to notice some acute phase changes such as parasitaemia and heart parasitism, both of which depend on the infecting strain [21–24]. In the present study, the animals primarily infected with the Y strain reached peak parasitaemia on Day 14 and these levels decreased abruptly on Day 21 of infection, whereas primary infection with the Colombiana strain showed low parasitaemia on the first days, with a substantial increase in the levels up to 21 days. These results are in accordance with the literature data [25].

Reinfected animals had much lower parasitaemia than primary infected animals, thus suggesting a possible protection conferred by the first infection, which was well demonstrated in Y/Y group, with undetectable parasitaemia 21 days after infection. It is in accordance with previous demonstrations that reinfected animals obtain immunological protection, thus leading to the reduction in parasitaemia and mortality [14, 26, 27].

Animals infected and/or reinfected with the Colombiana strain showed marked parasitism, whereas those reinfected with the Y strain did not have* T. cruzi* nests in any of the studied groups. Some authors argue that differences in the genetic composition of individual strains of* T. cruzi* would determine tissue tropism [28]. Classic studies had demonstrated that the Y strain is connected with reticulotropism and increased virulence in the acute phase of the infection, whereas the Colombiana strain is connected with cardiomyotropism and pathogenicity in the chronic phase [21–24].

Parasitism and inflammatory infiltrate were more severe in the heart of Colombiana infected and/or reinfected animals. A positive correlation between parasitism and the severity of myocarditis has been observed [29, 30], while aggravation of acute myocarditis seems to depend on the concentration and the quality of the exudate [31]. In this study, the inflammatory infiltrate was predominantly mononuclear in all groups. The severe inflammatory infiltrate observed in 66% of the animals of Col/Col group may be related to their higher mortality rate. Nonetheless, we did not observe increased fibrosis in these animals, which should be associated with the progression of cardiac insufficiency, as it is believed to lead Chagas disease patients to sudden death [32].

In Chagas disease, local immune response—represented by the inflammatory infiltrate—and systemic immune response are both responsible for the symptomatology and repercussions of the disease. In this study we analyzed the expression of TNF-*α*, IFN-*γ*, and IL-10, which are key cytokines in the anti-*T. cruzi* immune response. We observed that the levels of TNF-*α* in the cardiac tissue were elevated in Col and Col/Col groups and associated with a more severe inflammatory infiltrate and with the presence of* T. cruzi* nests. We believe that tissue parasitism in this group induced an increase in the expression of TNF-*α* *in situ*. TNF-*α* activates macrophages and, thus, the production of nitric oxide, leading to the destruction of intracellular parasites [6, 33, 34].

Similarly, when TNF-*α* production by spleen cells was analyzed, the Col and Col/Col groups had a significantly higher production than the Y or Y/Y groups, which could be explained by the fact that in the latter groups there were undetectable parasitism and mild inflammatory infiltrate, resulting in lower expression of TNF-*α* in the cardiac tissue and lower production by spleen cells* in vitro*. These results can be explained by a greater resistance of C57BL/6 mice to infection with the Y strain than to Colombiana strain of* T. cruzi* [35, 36]. Other experimental studies show that high levels of TNF-*α* in the acute phase seem to lead to cachexia and death, becoming an essential element of tissue inflammatory reaction [7, 37]. In the chronic phase of Chagas disease, TNF-*α* seems to be closely related to cardiac dysfunction owing to its negative inotropic effect, in both experimental models [38] and humans [39]. Furthermore, PBMCs of patients with chronic Chagas cardiopathy produce high levels of TNF-*α*, associated with higher expression of Fas and FasL, as well as with lymphocyte and myocardiocyte apoptosis [40, 41].

In the present study, the animals infected or reinfected with the Colombiana strain had a significantly higher expression of IFN-*γ*, both in the cardiac tissue and in spleen cells. Just like in the case of TNF-*α*, the groups with increased parasitism and cardiac inflammatory infiltrate expressed more IFN-*γ* in the cardiac tissue, in association with the most evident inflammatory infiltrate, particularly Col and Col/Col groups. Production of IFN-*γ* by spleen cells in Col/Col group had a mild decrease in relation to the group that was infected only with the Colombiana strain. This decrease may probably be due to cell recruitment to the cardiac tissue. Classic studies had already shown that IFN-*γ* and TNF-*α* are synergistic cytokines in the activation of macrophages and, hence, in the destruction of intracellular parasites [42].

We did not observe a difference in the expression of IL-10 in cardiac tissue among the studied groups. However, in stimulated culture, there seems to be a higher production in the groups infected and/or reinfected with the Colombiana strain. Interleukin-10 is a cytokine that modulates macrophage activity, being indirectly responsible for reduced IFN-*γ* production and for controlling the potential tissue damaging effects of activated macrophages [43]. In the present study, the groups that had the highest levels of TNF-*α* and IFN-*γ* also had the highest levels of IL-10. This increase may represent a compensatory mechanism aiming to control tissue damage caused by the local strong production of IFN-*γ* and TNF-*α*. Some studies on other intracellular pathogens demonstrated that the simultaneous raise of IFN-*γ* and IL-10 has a beneficial role in parasite control and in the prevention of tissue damage [7, 44, 45].* In vitro* studies, particularly on Chagas disease, have demonstrated that high IL-10 levels are capable of inhibiting the intracellular destruction of* T. cruzi* [46, 47]. Other studies using* il10* knockout mice showed that these animals have a more efficient control over the infection by* T. cruzi*, reducing parasitism levels with a significant increase in the secretion of IFN-*γ*, TNF-*α*, IL-12, and NO [48, 49]. However, animals with IL-10 deficiency succumb faster to* T. cruzi* infection mainly due to the uncontrolled activity of proinflammatory cytokines [49].

## 5. Conclusion

Our results suggest that mortality rates, tissue parasitism, inflammatory infiltrate, and expression of proinflammatory cytokines such as TNF-*α* and IFN-*γ*,* in situ* or* in vitro*, are differentially modulated by reinfections with* Trypanosoma cruzi* Y and Colombiana strains. This reinforces the need of control strategies involving parasite strain characterization in endemic areas for Chagas disease.

## Figures and Tables

**Table 1:** Amastigotes nests, inflammatory infiltrate, and fibrosis in cardiac tissue.

Groups	Amastigotes nests (%)	Inflammatory infiltrate %			Fibrosis (% area ± SEM)
		Mild	Moderate	Severe	
Col	16.6	0	100	0	0.70 ± 0.13
Col/Col	33.3∗	0	33.3	66.6∗	0.28 ± 0.04
Col/Y	0	50	50	0	0.84 ± 0.79
Y	0	100	0	0	0.31 ± 0.09
Y/Y	0	100	0	0	0.56 ± 0.14
Y/Col	10	0	100	0	0.37 ± 0.10
Control	—	—	—	—	0.39 ± 0.17

The qualitative variables were expressed in percentage, and the associations between them were analyzed using Chi-square test (*χ* ^2^), *P < 0.005. The percentage of fibrosis in the cardiac tissue was analyzed using ANOVA test followed by Tukey's multiple comparison test. ∗Significant differences among Col group versus Col/Col versus Col/Y versus control group.
